# In-Situ Optimization of an Optoelectronic Reservoir Computer with Digital Delayed Feedback

**DOI:** 10.1021/acsphotonics.5c01056

**Published:** 2025-07-11

**Authors:** Fyodor Morozko, Shadad Watad, Amir Naser, Antonio Calà Lesina, Andrey Novitsky, Alina Karabchevsky

**Affiliations:** † School of Electrical and Computer Engineering, 26732 Ben-Gurion University of the Negev, Beer Sheva, 8410501, Israel; ‡ Department of Physics, Lancaster University, Lancaster LA1 4YB, United Kingdom; § Hannover Centre for Optical Technologies, Institute for Transport and Automation Technology, and Cluster of Excellence PhoenixD, 26555 Leibniz University Hannover, Hannover, 30167, Germany; ∥ Belarusian State University, Minsk, 220030, Belarus

**Keywords:** reservoir computing, neuromorphic computing, physical computing, optoelectronic oscillator, in situ optimization

## Abstract

Reservoir computing (RC) is a powerful computational framework that addresses the need for efficient, low-power, and high-speed processing of time-dependent data. While RC has demonstrated strong signal processing and pattern recognition capabilities, its practical deployment in physical hardware is hindered by a critical challenge: the lack of efficient, scalable parameter optimization methods for real-world implementations. Traditionally, RC optimization has relied on software-based modeling, which limits the adaptability and efficiency of hardware-based systems, particularly in high-speed and energy-efficient computing applications. Herein, an in situ optimization approach was employed to demonstrate an optoelectronic delay-based RC system with digital delayed feedback, enabling direct, real-time tuning of system parameters without reliance on external computational resources. By simultaneously optimizing five parameters, normalized mean squared error (NMSE) values of 0.028, 0.561, and 0.271 are achieved in three benchmark tasks: waveform classification, time series prediction, and speech recognition, outperforming simulation-based optimization with NMSEs 0.054, 0.543, and 0.329, respectively, in two of the three tasks. This method enhances the feasibility of physical reservoir computing by bridging the gap between theoretical models and practical hardware implementation.

## Graphical abstract

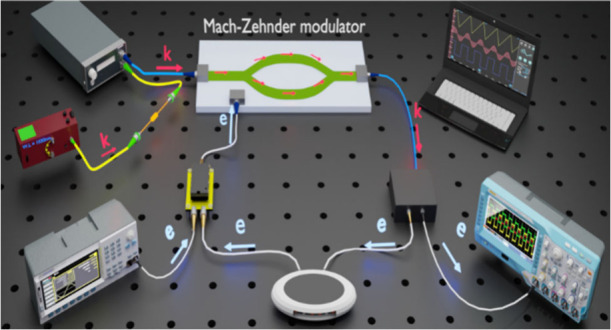

## Introduction

In the realm of machine learning, Artificial Neural Networks (ANNs) are powerful algorithms inspired by biological neural systems consisting of interconnected neurons organized in layers, granting them a remarkable ability to learn and excel in tasks like pattern recognition and decision-making.^1^ The past decade has seen explosive growth in the exploration and exploitation of these algorithms due to the emergence of Deep Neural Networks, consisting of multiple layers.^2^

ANNs have an inherently analogue nature, so their implementation within traditional von Neumann/Turing architecture is suboptimal regarding computation speed and energy consumption. Therefore, the ever-growing utilization of ANNs as well as the approaching limits of Moore’s law motivate the exploration of analogue (or physical) computing. Optical computing has emerged as a promising candidate for reshaping data processing through the inherent advantages of parallelism, high bandwidth, low noise, and rapid signal propagation.^3–11^

Among ANN architectures, reservoir computing (RC), a special case of Recurrent Neural Network (RNN) relying on randomized rather than trained recurrent connections (weights) stands out as a highly attractive paradigm.^12^ The randomized nature of weights allows avoiding the notorious issue of exploding/vanishing gradient in RNNs training and solves the overfitting problem, improving model generalization to unseen data.^13,14^ Furthermore, the fixed topology of RC makes this architecture highly suitable for physical implementations that emerged first in the electronic platform and soon extended to diverse physical platforms, particularly, to optics and optoelectronics.^15–20^

In the heart of reservoir computing is a nonlinear network of randomly interconnected nonlinear dynamical nodes called a reservoir. These nodes receive the input signal ***u*** to be processed via the input connectivity matrix *W* ^*I*^. The evolution of the reservoir’s state ***x*** thus can be described as.^12,21^

1 $$x\left(n+1\right)=f\left(\beta Wx\left(n\right)+\rho W^{I}u\left(n+1\right)\right)$$

where β and ρ are the feedback and input scaling, respectively. *n* is the discrete-time, *f* is a nonlinear activation function, *W* is the reservoir connectivity matrix. The length of the vector ***x*** is the size of the reservoir, which is equal to the number of neurons. The reservoir’s response, corresponding to the network’s reaction to the input signal, is evaluated at the read-out nodes *y*_out_ as

2 $$y_{\mathrm{out}\;}=W^{R}x\left(n\right)$$

where *W* ^*R*^ is the trainable readout matrix. In eq 2, a bias term can be included. Hence, the hallmark feature of RC: the nonlinearity performing information processing is encapsulated in the reservoir, while the training comprises a linear regression problem to find the readout matrix *W* ^*R*^. Figure 1 schematically depicts the structure of a generic reservoir computing (RC) network.

**Figure 1:**
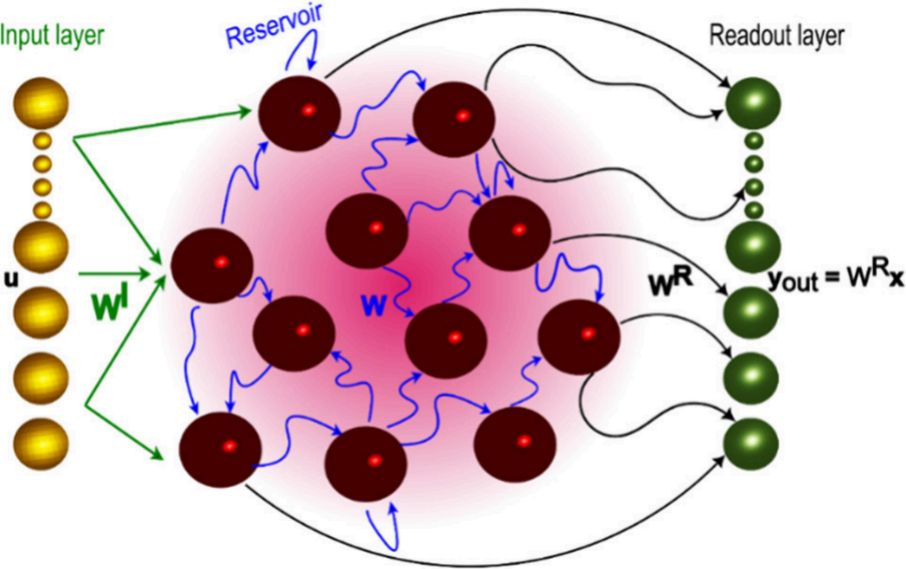
Generic reservoir computing architecture. The depicted layout consists of distinct components: an input layer (bronze spheres) responsible for receiving external data, a reservoir (ruby spheres) featuring randomized fixed connections, and a linear readout layer (green spheres).

In 2011 Appeltant et al. realized that RC can be implemented with a single physical node in delayed feedback systems.^21^ Thereby implementation of RC in delayed feedback systems simplifies physical implementation while demonstrating excellent efficiency in processing sequential data, making this architecture a compelling choice for tasks involving temporal dependencies and time-series analysis.^1–6^

Computational use of delayed-feedback systems leverages their capacity to exhibit complex dynamical behavior, despite being governed by seemingly simple equations. This hidden complexity was elucidated through the two-dimensional spatiotemporal representation introduced by Arecchi et al.^23^

Optoelectronic oscillators—photonic delay systems composed of electro-optic modulators and photodetectors arranged in a feedback loop with an added delay—serve as a prominent example. These systems initially attracted interest in the study of nonlinear dynamics due to their ability to exhibit rich, complex behavior, which arises from the sinusoidal transmission characteristics of the electro-optic modulator.^24–26^ Particularly, optoelectronic oscillators, upon the tuning of parameters, exhibit a transition from stable to periodic and from periodic to chaotic dynamics known as the Hopf bifurcation.^27,28^ Shortly after the revival of neural networks, optoelectronic oscillators were recognized as a particularly suitable platform for physical computing, including RC, due to high modulation bandwidth, robustness, and high controllability of parameters.^15,17,19,29^ Because electro-optic modulators can be fabricated using a variety of material platforms, such as electro-optical crystals, their integration into neural network architectures broadens the material landscape of modern computing, which remains predominantly reliant on silicon.^30^

Apart from the fixed or trainable weights, any machine learning model, including RC, possesses a set of external configuration variables that are set before training. In the machine learning vocabulary, such variables are called hyperparameters.^31^ In the context of RC reservoir size, input and feedback scaling appearing in eq 1 are examples of hyperparameters. Owing to the fixed nature of internal weights and the linearity of the readout layer, which uniquely defines the readout weights, all actual design degrees of freedom of an RC model are encompassed in hyperparameters, making hyperparameter optimization crucial for achieving good accuracy within RC.^32^

Considering physical RC, hyperparameter optimization, if done, is typically performed using a simulation model.^17,29^ The parameters identified in the model optimization are then applied to the physical system. However, this approach suffers from limited scalability, first, due to the necessity of characterization of all the system components; second, by not benefiting from hardware acceleration at the optimization stage, which obscures the advantages of using physical computing; third, model-based optimization requires modification of the model if any change is introduced into the architecture. On the other hand, if hyperparameters of a physical RC system can be set programmatically as well as the corresponding performance can be programmatically evaluated, it is possible to perform the optimization *in situ, i.e.,* using the system itself, and to avoid the model-related problems.

The choice of RC is motivated by its unique compatibility with delay-based physical systems, particularly optoelectronic oscillators with feedback loops. Unlike conventional neural network paradigms such as multilayer perceptrons or convolutional neural networks, RC requires training only at the readout layer, while the reservoir dynamics remain fixed. This dramatically simplifies the training process and enables direct implementation in hardware without relying on energy-intensive backpropagation algorithms.

Our hardware platform—a fiber-based optoelectronic loop comprising a Mach–Zehnder modulator and FPGA-based delay—naturally supports the temporal dynamics required for RC. Specifically, the time-multiplexed representation of virtual nodes along the delay line maps seamlessly onto the reservoir architecture. In contrast, other neural architectures would demand large-scale matrix multiplications and iterative gradient updates, which are difficult to realize in analog or hybrid analog-digital systems and often impose significant energy and memory overhead.

Therefore, RC not only aligns with the physical characteristics of our platform but also enables real-time, low-power signal processing, making it a particularly suitable and efficient computational paradigm for this implementation.

In this study, we implement an optoelectronic reservoir computing (RC) system featuring digitally programmable delay feedback. The use of digital delay is motivated by its broad programmability, enabling direct control over key hyperparameters, including delay time, and facilitating in situ optimization. Through multiple benchmark tasks, we demonstrate that in situ optimization not only achieves state-of-the-art accuracy but also eliminates the need for simulation-based modeling.

## Results

### Experimental Setup

The experimental setup is illustrated in Figure 2a, which provides an artistic rendering of the system. Additionally, Figure 2c presents a photograph of the actual physical system as implemented in the laboratory. We have used a continuous-wave (CW) laser at a wavelength of 1550 nm (KLS1550, Thorlabs) as a monochromatic light source. The laser was connected through a fiber polarization controller (PC1100, Fiberpro) to an Erbium-doped fiber amplifier (EDFA) (EDFA100S, Thorlabs) working in a saturated regime. The EDFA was used to increase the maximum optical power level, while the variable optical attenuator (VOA) was used to adjust the optical power level in the reservoir. The attenuated optical signal was modulated with a Mach–Zehnder modulator (MZM) (LN81S-FC, Thorlabs) produced in X-cut lithium niobate. The modulated optical signal was detected and amplified with an InGaAs photodetector (PDB450C, Thorlabs) with an embedded switchable gain trans-impedance amplifier. The voltage output from the photodetector was sent to Moku:Go (Liquid Instruments), an FPGA-based instrument that implements a delay line and data acquisition. We stress that the short-time memory property is introduced in the system by the delayed feedback, while the nonlinearity is introduced by the sinusoidal transmission characteristic of the MZM. Moku:Go was also used as a programmable voltage source controlling the phase bias of the MZM and attenuation of the VOA. The output of Moku:Go was mixed with the input signal synthesized with an arbitrary waveform generator (33220A, Agilent). To drive the MZM, the mixed signal was amplified with a driver circuit based on Texas Instruments’ LM7171 operational amplifier. To ensure robustness and minimize environmental drift, the entire system was housed in a temperature-stabilized environment. The FPC was used to consistently align the input light along the MZM’s slow axis, mitigating its inherent polarization sensitivity. Additionally, the EDFA was operated in a saturated regime to stabilize optical power fluctuations. All optimization procedures were conducted in situ under these real-time conditions, which means the reported performance inherently accounts for potential fluctuations in temperature and polarization. This approach reinforces the system’s reliability and reproducibility under practical operating conditions.

**Figure 2:**
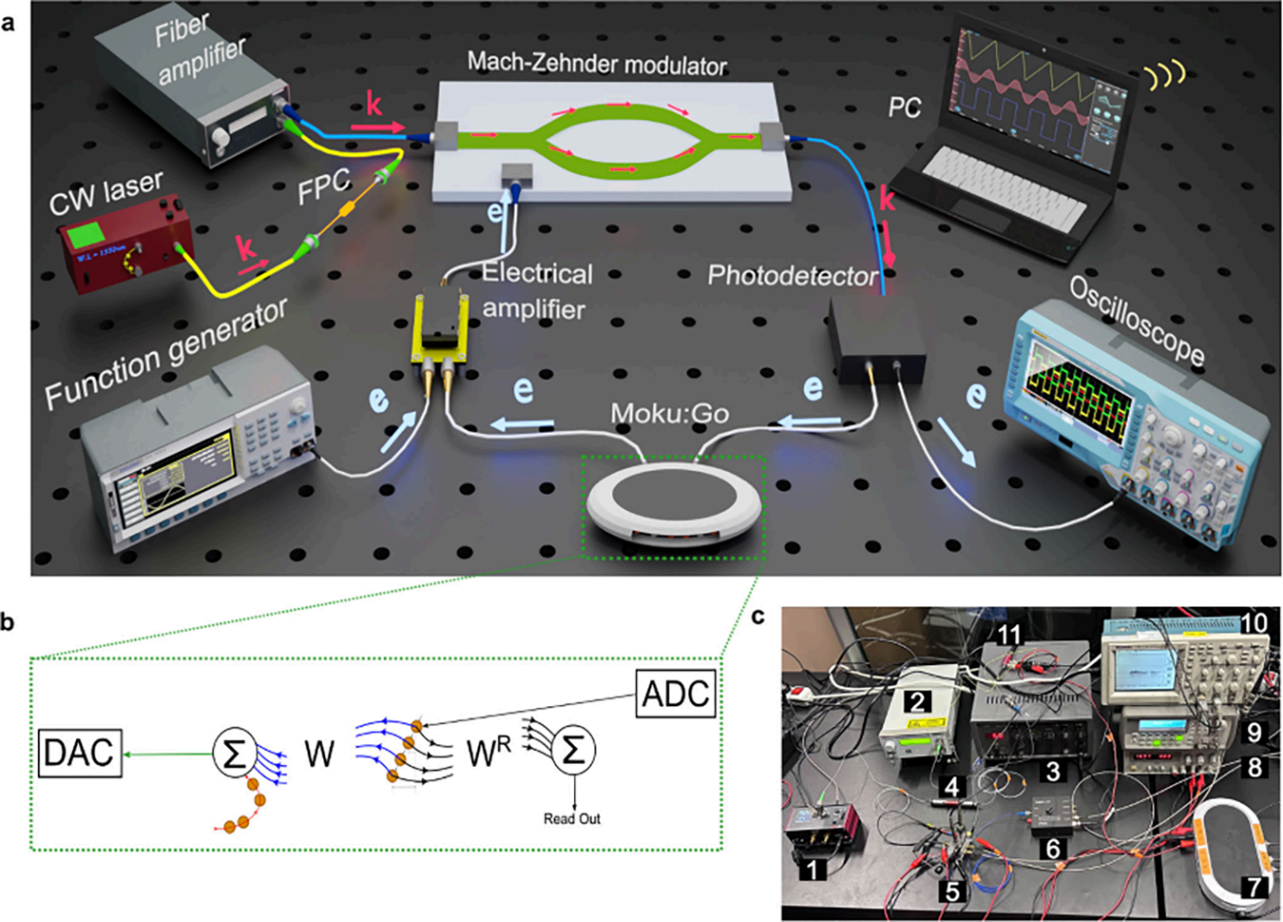
**a** An artistic impression of the experimental setup. A continuous wave (CW) laser beam is directed into the fiber polarization controller (FPC), which aligns the light polarization with the slow axis of the modulator. Subsequently, the laser beam is coupled to a fiber amplifier to maintain the system’s stability. This amplified laser light is modulated with a Mach–Zehnder modulator (MZM), whose sinusoidal transmission function introduces nonlinearity into the reservoir. The modulated light is detected by a photodetector, delayed with Moku:Go’s FPGA-based delay, amplified, and sent to the modulation input of the MZM, forming a closed loop. The external signal is mixed with the delayed feedback. Note: the components are out of scale for visualization. **b** Schematics of the digital delay and data digitizer implemented in the Moku:Go component: delayed feedback implements the reservoir’s connectivity matrix *W*, while the digitized reservoir states are weighted with the readout matrix *W* ^*R*^. **c** Photograph of the experimental setup, components are labeled as follows: 1. continuous-wave (CW) laser, 2. Erbium-doped fiber amplifier (EDFA), 3. A function generator producing a trigger signal. 4. fiber polarization controller (FPC), 5. Mach–Zehnder modulator (MZM) with electrical driver, 6. photodetector, 7. Moku:Go, 8. power supply for the electrical driver, 9. arbitrary waveform generator (AWG), 10. oscilloscope, 11. variable optical attenuator (VOA).

### Reservoir Performance

Here, we present the results of in situ optimization of our experimental RC system in three benchmark tasks and compare them to the simulation. In each task, we simultaneously optimized five parameters: gain G, phase bias Φ_0_, input scaling ρ, delay time τ, and regularization parameter λ. For the optimization, a Bayesian algorithm was employed in refs 32, 41, and 39 along with random search.

#### Sinusoidal vs Rectangular Waveform Classification

As a first experiment, we have trained the reservoir to distinguish sinusoidal from rectangular waveforms following Paquot et al.^17^ Departing from ref 17, we varied the frequency of the waveforms to increase the complexity of the task. For the training, the data set consisting of 20 waveforms was split equally into train and test sets. Figure 3 presents the simulated and experimental reservoir performance on the test data set. As illustrated in the figure, both the simulated and experimental reservoirs successfully achieved perfect classification of the waveforms. The experimental system, however, exhibited a slightly more stable readout signal, resulting in NMSE outperforming simulation by almost a factor of 2:0.028 and 0.054, respectively.

**Figure 3:**
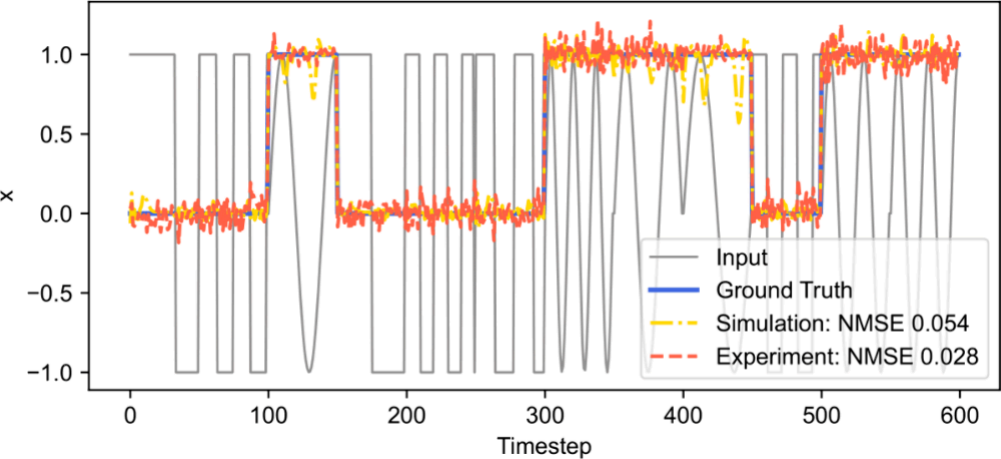
Sinusoidal vs rectangular waveform classification task. The solid gray curve represents the input signal (sinusoidal and rectangular waveforms), and a solid blue curve represents the target response (*x* = 1 for sinusoidal, *x* = 0 For a rectangular waveform), the dash-dotted yellow and orange dashed curves represent the readout of simulated and in situ-optimized experimental reservoirs, respectively. Experimental reservoir settings: input scaling ρ = 0.19, net gain *G* = 0.39, phase bias Φ_0_ = 0.67π, delay τ = 0.27*T*, regularization parameter λ = 1.4 × 10^–3^.

The optimal parameter settings obtained *in situ* were: input scaling ρ = 0.19, net gain *G* = 0.39, phase bias Φ_0_ = 0.67π, delay τ = 0.27*T*, regularization parameter λ = 1.4 × 10^–3^.

#### NARMA10 Time Series Recovery

For a second benchmark, we trained the reservoir to predict time series generated by the Nonlinear Auto Regressive Moving Average (NARMA) model,^13,17^ a popular benchmark task in the RC literature.

We used a NARMA model of order ten driven by the equation

3 $$y\left(n+1\right)=0.3y\left(n\right)+0.05y\left(n\right)\left(\overset{9}{\underset{i=0}{\sum }}y\left(n-i\right)\right)+1.5u\left(n-9\right)u\left(n\right)+0.1$$

analogous to the model in refs 13, 17, and 39 .

The total length of the data set was 8000 steps; the data set was randomly split into train and test sets, each 4000 steps long.

Reservoir performance in this task is presented in Figure 4. One observes almost identical behavior of simulated and experimental reservoirs and similar NMSE of 0.543 vs 0.561, respectively.

**Figure 4:**
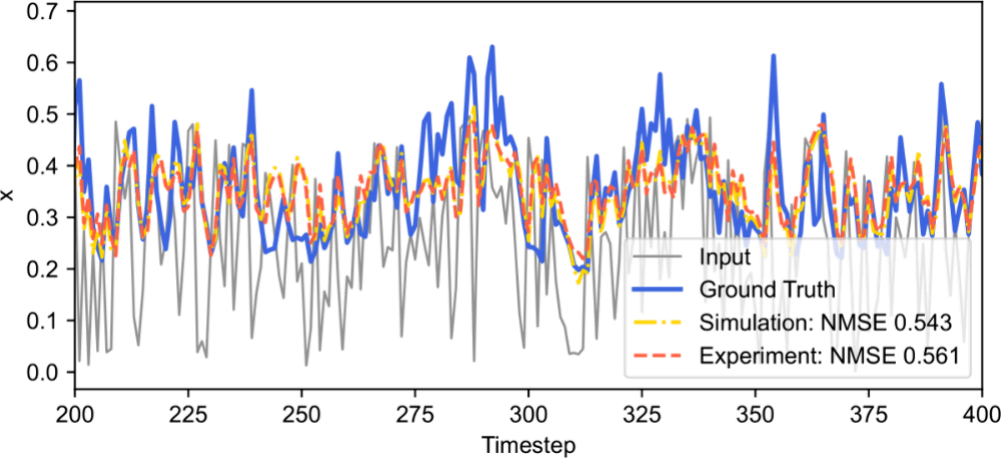
NARMA10 time series recovery. The solid blue curve represents the input signal (white noise), the solid blue curve represents the time series governed by the NARMA10 model (eq 3), the dashed-dotted yellow and dashed orange curves represent the readout of simulated and in situ-optimized reservoirs, respectively. Experimental reservoir settings: input scaling ρ = 0.33, net gain *G* = 0.7, phase bias Φ_0_ = 0.68π, delay τ = 0.49*T*, regularization parameter λ = 5 × 10^–3^.

The optimal settings of the reservoir were found to be ρ = 0.33, *G* = 0.7, Φ_0_ = 0.68π, τ = 0.49*T*, and λ = 5 × 10^–3^ for the reservoir of size 50. The performance of the best reservoir configuration for this task was found to be 0.534 and 0.731 in terms of NMSE and NRMSE, respectively, at the reservoir size of 50. Our NMSE is considerably higher than that in ref 17 (0.168) but is within the range of NMSEs reported in ref 39 (∼0.04–0.64) for simulated reservoirs of the same size and variable delay to clock cycle ratio. We attribute higher error in this case to the noise arising from the quantization error introduced by digital delayed feedback

#### Japanese Vowels Classification

To assess the speech recognition capability of our system, we tested it on the Japanese Vowels data set.^34^ This data set contains a 640-time series of 12 Mel-frequency cepstrum coefficients (MFCCs) taken from recordings of nine speakers uttering a Japanese vowel. The task is to classify the recordings by the speaker’s identity.

The optimal settings of the reservoir were found tobe ρ = 0.47, *G* = 0.52, Φ_0_ = 0.44π, τ = 0.35*T*, and λ = 3 × 10^–7^ for the reservoir with 50 nodes. The results are presented in Figure 5. In this task, the best NMSE was found to be 0.271 with the corresponding word error rate (WER) of 6.5%, comparable to the result in ref 22 and outperforming ref 35 with 18.5% WER.

**Figure 5:**
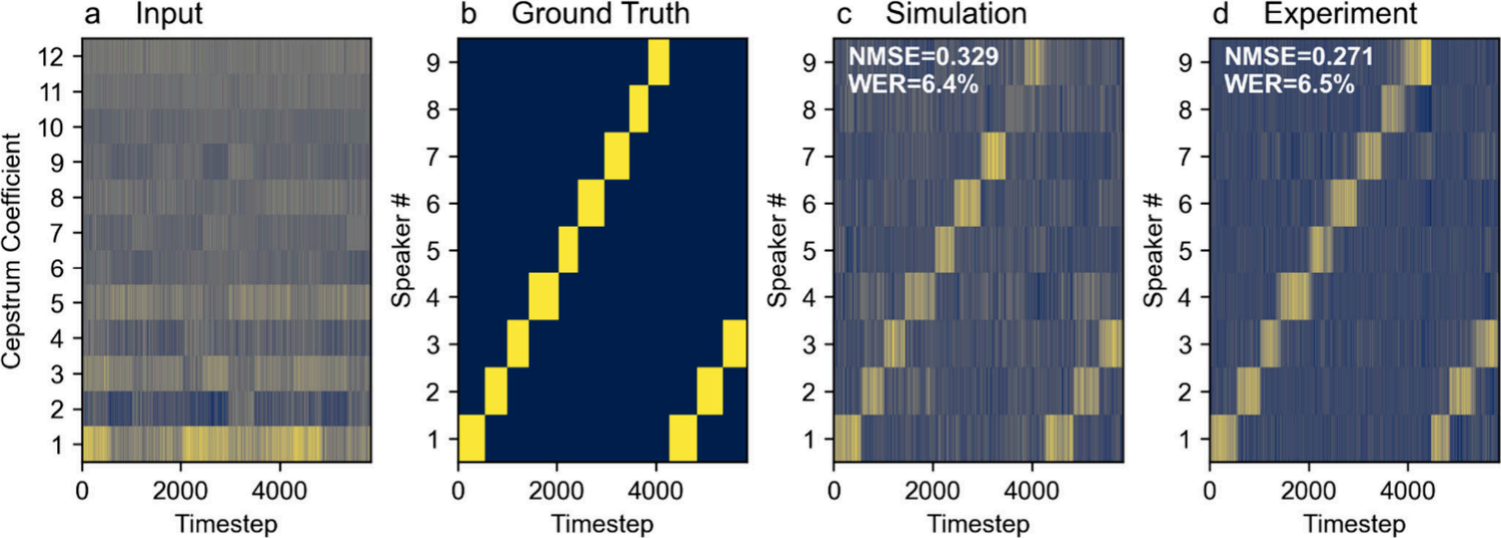
Japanese vowels classification task. multiplexed waveforms of **a** input data, **b** ground truth, **c–d** simulated and experimental reservoirs’ readouts. Reservoir settings: input scaling ρ = 0.47, net gain *G* = 0.52, phase bias Φ_0_ = 0.44π, delay τ = 0.35*T*, regularization parameter λ = 3 × 10^–7^.

#### Effect of the Parameters on the RC Accuracy

As we discuss in the section Delayed Feedback Tuning Using FPGA in Materials and Methods, it is predicted that the RC performance drastically depends on the delay to clock cycle interplay and exhibits an important degree of freedom.^33,36^ We have performed optimization of the five parameters of the reservoir as discussed in the section In Situ Reservoir Optimization in Materials and Methods. Curiously, by performing *in situ* optimization, we have confirmed this theoretically predicted effect experimentally. Figure 6 shows the performance distribution across reservoir parameter settings in the NARMA10 task.

**Figure 6:**
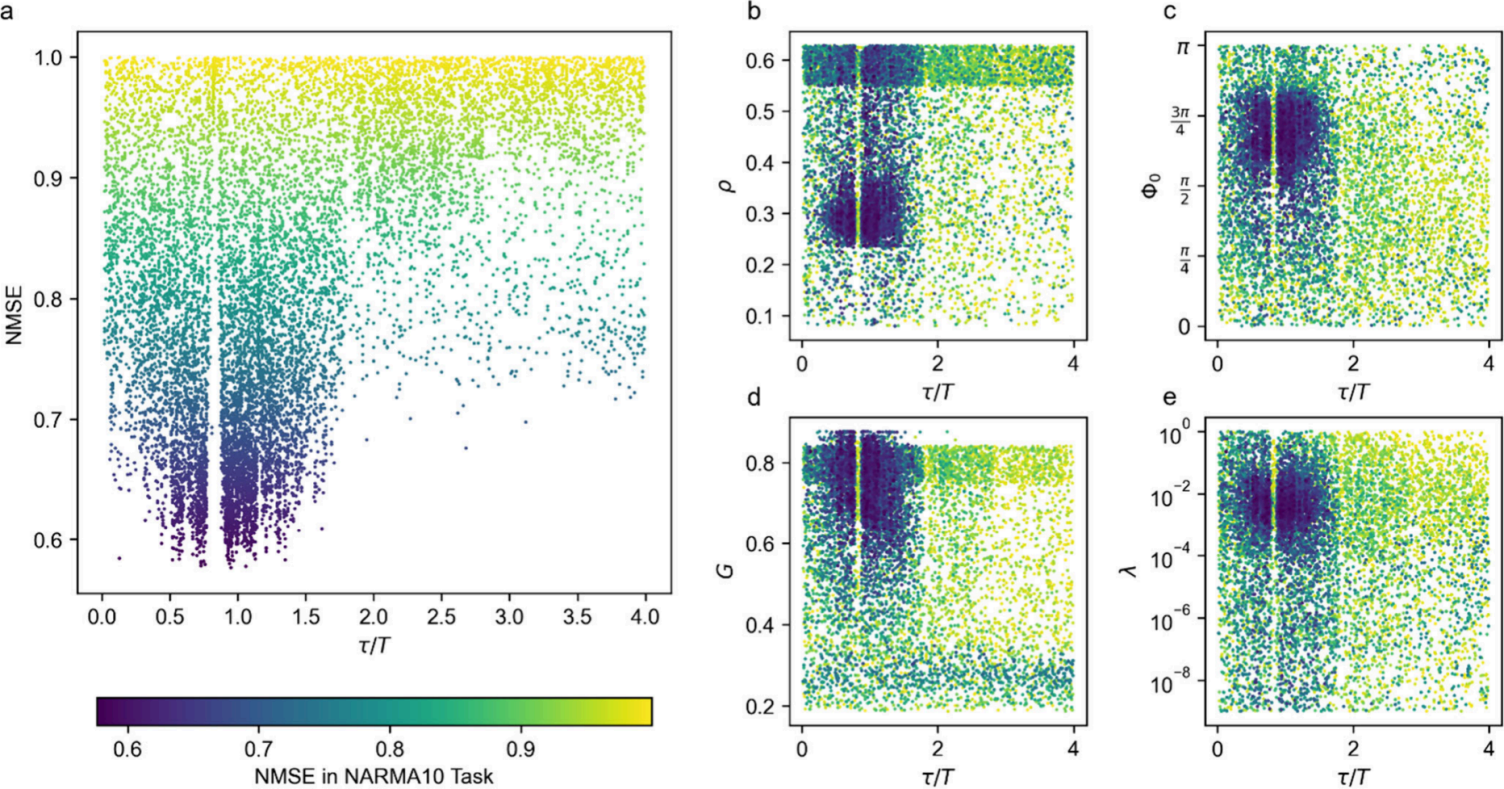
*In situ* optimization results. Normalized mean square error (NMSE) in NARMA10 task as a function of **a** delay to clock cycle ratio τ/*T* and **b–e** τ/*T* together with another parameter of the reservoir: **b** τ/*T* and input scaling ρ, **c** τ/*T* and phase bias Φ_0_, **d** τ/*T* and net gain *G*, **e** τ/*T* and regularization parameter λ. Each point in the plots corresponds to a tested reservoir parameter setting.

In addition to confirming the importance of the delay-to-clock ratio, our analysis reveals varying sensitivity levels for the other four parameters. Input scaling (ρ) and phase bias (Φ_0_) exhibit single, well-defined optima, indicating that small deviations from these values lead to noticeable NMSE degradation. The gain (G) exhibits a broader optimal range, although multiple local minima suggest sensitivity to the reservoir’s dynamic regime. The regularization parameter (λ), in contrast, has a smoother influence, impacting generalization but with less drastic NMSE changes. These observations are consistent across the optimization traces in Figure 6b–e and highlight that while all five parameters influence performance, $\frac{\tau }{T}$ , ρ, and Φ_0_ are particularly critical for achieving low NMSE.

The points on the graph correspond to the RC configurations tested during the optimization. Parameter configurations were sampled using a Bayesian optimization algorithm and additionally by random sampling to explore the parameter space landscape.

The best performance is observed within the range of ratios τ/*T* ∈ [0,2] with multiple peaks and drops in NMSE. The largest peak corresponds to the resonance τ/*T* ≈ 1 but is shifted to the left. We attribute this shift of the main peak in Figure 6 to the latency of the FPGA, resulting in slightly increased actual τ. Noteworthy, performance boost at nonresonant τ/*T* ratios do not depend on any other reservoir parameters in accordance with ref 37. This result is, to our knowledge, the first experimental verification of the detrimental effect of delay time on clock cycle resonances made in refs 36, 37 and discussed in ref 33. Table 1 summarizes the performance metrics (NMSE and WER) achieved by our system across benchmark tasks and compares them with results from prior experimental RC implementations.

**Table 1:** Comparison of Performance Metrics (NMSE and WER) for Benchmark Tasks Across Prior Experimental Delay-Based RC Systems and the Present Work

benchmark task	metric	ref 17	ref 33	ref 35	This Work
Waveform Classification	NMSE	∼0.05	–	–	0.028
NARMA10 Time Series Prediction	NMSE	0.168	0.04–0.64 (sim.)	–	0.561
Japanese Vowel Recognition	WER	–	–	5%	6.5%
Japanese Vowel Recognition	NMSE	–	–	–	0.271

## Discussion

To conclude, we have demonstrated model-free in situ optimization of a physical reservoir computing system implemented in an optoelectronic oscillator with digitally programmable delayed feedback. By simultaneously tuning five key system parameters—input scaling (ρ), gain (G), phase bias (Φ_0_), delay time (τ), and readout regularization parameter (λ)—using a Bayesian optimization algorithm, we achieved state-of-the-art accuracy across three benchmark tasks involving pattern recognition and time series prediction.

This work highlights the practical advantages of in situ optimization in physical computing. First, it eliminates the need for labor-intensive numerical modeling that requires a comprehensive characterization of all system components and environmental factors. Second, it leverages the inherent speed and energy efficiency of physical systems during the optimization process itself. Third, it offers a scalable solution for complex architectures where simulation is impractical due to stiff governing equations or limited parameter access. Importantly, our findings experimentally confirm that the delay-to-clock-cycle ratio is a critical factor influencing system performance, corroborating recent predictions from numerical studies. These results underscore in situ optimization as a powerful and practical approach for advancing the deployment of physical reservoir computing systems in real-world applications.

Beyond benchmarking accuracy, our system also offers significant advantages in terms of computational speed and energy efficiency when compared to traditional digital and hybrid reservoir computing (RC) platforms. The use of high-speed analog signal propagation and FPGA-based feedback enables real-time operation at sampling rates up to 3.906 MHz. This corresponds to a substantial throughput advantage over digital RC systems, which are often limited to kilohertz sampling rates due to CPU or GPU bottlenecks. Additionally, the *in situ* optimization method eliminates the need for energy-intensive iterative simulations typically required in software-based systems. By conducting the optimization directly within the physical system, we bypass large-scale numerical computation, leading to a reduction in both computational load and energy use during training. This aligns with the goals of analog physical computing, where efficiency is achieved through hardware-native adaptation. Our NMSE values, benchmarked against prior works,^17,33,35^ further strengthen the competitive performance of our approach in both accuracy and runtime characteristics.

The *in situ* optimization approach demonstrated here is also inherently scalable to larger reservoir sizes and more advanced multiplexing strategies. In our delay-based architecture, the number of virtual nodes is determined by the ratio between the clock cycle (T) and the sampling interval (θ), both of which are tunable via the FPGA configuration. This allows the number of nodes to be significantly increased into the hundreds, while maintaining real-time operation, as our system supports sampling rates up to 3.906 MHz. Since the readout remains linear, training complexity grows only linearly with the number of nodes, and the optimization of the readout weights remains computationally efficient via standard linear regression. Furthermore, the framework is compatible with alternative architectures such as space-multiplexed and hybrid delay-reservoir systems. These systems may incorporate multiple feedback loops, spatial input masks, or distributed nonlinear elements, all of which can be addressed by extending the optimization parameter space. Because optimization is performed directly on the system output and driven by task-specific performance metrics such as NMSE, the approach remains robust against practical challenges like device imperfections, thermal drift, and parameter crosstalk. This makes it particularly promising for scaling up physical reservoir computing systems toward high-dimensional neuromorphic platforms.

## Materials and Methods

### Training of the Reservoir

Training of the reservoir was performed by finding the weights (elements of the matrix *W* ^*R*^) minimizing the mean squared error (MSE) ∥ ***y***_*t*_ – *W* ^*R*^ ***x*** ∥^2^ between the target output ***y***_*t*_ and reservoir’s readout given by eq 2 on a training data set using linear regression. To avoid overfitting, a regularization term was added to the MSE ∥ ***y***_*t*_ – *W* ^*R*^ ***x*** ∥^2^ + ∥*λW* ^R^ ***x*** ∥^2^, with the regularization constant λ also referred to as the ridge constant. Due to the linearity of the readout layer, it is possible to find the readout weights by simple matrix inversion as

4 $$W^{R}=YX^{T}{\left(XX^{T}+\lambda I\right)}^{-1}$$

where *X* and *Y* matrices are obtained by column-wise concatenation of all reservoir states ***x*** (*n*) with *n* = 0, 1, ..., *N* – 1 and all target outputs ***y***_*t*_(*n*) with *n* = 0, 1, ..., *N* – 1, respectively, where *I* is the identity matrix.^38^ An important feature of eq 4 is that it possesses a unique solution in contrast to the gradient descent algorithm, whose solution depends on the choice of the initial point.

The performance of the reservoir is evaluated by measuring the error on a validation data set not seen during training. One of the commonly used error metrics for evaluation is the normalized mean squared error (NMSE) defined as

5 $$\mathrm{NMSE}=\frac{∥y-y^{\prime }{∥}^{2}}{\sigma_{y}^{2}}$$

where ***y*** and ***y*** ^**’**^ are the target and the actual response on the validation data set, respectively, while σ_*y*_^2^ is the variance of the target response.^40^ Another popular metric is the normalized root-mean-square error (NRMSE), which is equal to the square root of NMSE.

### Time Multiplexing in Delay-Based Reservoirs

To relate the temporal dynamics of a delay-based reservoir with the evolution eq 1, time multiplexing is performed: components of the reservoir’s state vector ***x*** (*n*)  ≡ *x* ^*i*^(*n*) are encoded in values of the reservoir’s state variable *x* sampled at evenly spaced instants of time during a clock cycle *T* so that the *i*-th component at the *n*-th cycle corresponds to the time instant *t*_*i*_ = *iθ* + *nT*

6 $$x\left(n\right)\equiv x^{i}\left(n\right)=x\left(i\theta +nT\right)$$

where *i* = 0, ...,*k* – 1, θ = *T*/*k* is the neuron’s temporal separation with *k* being the size of the reservoir. Time multiplexing is closely related to the spatiotemporal representation of the delayed feedback systems.^23^

To map the input signal onto the internal space of the reservoir, input masking is performed by applying a sample-and-hold operation to the input and multiplying by a periodic piecewise-constant function with the period *T*.^21,29^ For establishing interactions between different neurons in successive layers (expressed by off-diagonal entries in the matrix *W* of eq 1, several approaches to time multiplexing exist: in ref 15 delay and clock cycle were synchronous τ = *T* while an introduced low-pass transient characteristic caused neighboring neurons to interact, in ref 16 analogously, delay and clock cycle were synchronized, but multiple fractional delays were introduced. It was observed by Rodan et al.^13^ and first exploited in hardware by Paquot et al.^17^ that desynchronization of the clock cycle and delay time readily leads to a nontrivial reservoir topology by causing different neurons in the successive recurrent layers to interact. Specifically, in refs 17 and 19 the delay time was set to τ = *T* + θ providing a minimum complexity network structure suggested in ref 13. The structure of a delay-based RC is schematically shown in Figure 7.

**Figure 7:**
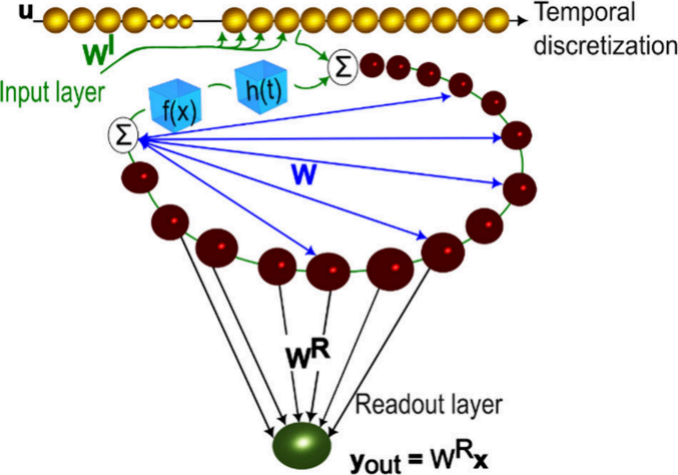
Delayed feedback-based reservoir computing *f*(*x*) is the activation function performing the nonlinear transformation exhibited by the element, and *h*(*t*) is the impulse response. *W*, *W* ^*I*^, and *W* ^*R*^ are, respectively, reservoir, input, and readout connectivity matrices.

### Model of Optoelectronic Oscillator

We consider an optoelectronic oscillator comprising a continuous wave light source, a Mach–Zehnder modulator (MZM), and a photodetector whose output is connected to the modulation port of the MZM through a delay line. Dynamics of such an optoelectronic oscillator can be described by the Ikeda model^24,25,27,28^ according to which voltage *V* at the modulation port of MZM follows the delay-differential equation follows:

7 $$V\left(t\right)+T_{R}\frac{dV}{dt\left(t\right)}=G^{*}P\left[V\left(t-\tau \right)\right]$$

where *P*[*V*] is the power transmitted by the MZM and received by the photodetector, τ is the delay time, G* is the voltage gain of the photodetector, and *T*_*R*_ is the response time of the system. The transmission characteristic *P*[*V*] of the Mach–Zehnder modulator is given by^26–28^

8 $$P\left[V\right]=\frac{1}{2}P_{\max}\left(1+M\sin\left(\frac{\pi \left(V+V_{B}\right)}{V_{\pi }}+\phi \right)\right)$$

where *P*_max_ is the total optical power in the system, *M*, *V*_π_, and ϕ are the modulation depth, half-wave voltage, and intrinsic phase of the MZM, respectively, and *V*_B_ is the bias voltage at the bias port of the MZM. The bias voltage *V*_***B***_ directly influences the MZM’s nonlinear response by shifting the argument of the sinusoidal transmission function. Specifically, it defines the phase offset $\Phi_{0}=\phi +\frac{\pi V_{B}}{V_{\pi }}$ , which sets the operating point of the modulator along the sine curve. By varying *V*_B_, the system’s operating point can be positioned at regions of the curve with different local slopes, ranging from nearly linear to strongly nonlinear regimes. This control is essential for ensuring the nonlinearity injected into the reservoir. Operating near points of maximum slope enhances sensitivity and enables complex transformations, while operating near flatter regimes of the sine curve can lead to reduced dynamic range or signal saturation. As is seen from eqs 7–8) the sinusoidal transmission characteristic renders the optoelectronic oscillator a nonlinear dynamical system. If the response time *T*_R_ of the photodetector and its subsequent amplifier is significantly shorter than the delay time τ (*T*_*R*_ ≪ τ), the derivative term in eq 7 can be neglected. This allows the system to be accurately described by the discrete-time difference equation^26^

9 $$V\left(t\right)=G^{*}P\left[V\left(t-\tau \right)\right]$$

Operating in this fast-response regime ensures that the photodetector does not act as a low-pass filter, thereby preserving the temporal resolution and internal dynamics of the reservoir. A slower photodetector would introduce signal smearing, reduce the system’s memory capacity, and impair its ability to perform temporally sensitive tasks such as NARMA10 prediction.

While analyzing *V*(*t*) at discrete time steps *t* = *t*_0_ + *nτ*. Introducing the dimensionless state variable *x* = *V*/*V*_π_, performing time multiplexing according to eq 6 and taking eqs 8–9) into account, we convert eq 7 into a generic RC evolution eq 1 as

10 $$x\left(n+1\right)=\frac{G}{2}\left(1+M\sin\left(\beta Wx\left(n\right)+\rho W^{I}u\left(n+1\right)+\Phi_{0}\right)\right)$$

where the external signal ***u*** is introduced, Φ_0_ = ϕ + *πV*_*B*_/*V*_π_ is the phase bias, β and ρ are the feedback and input scaling, respectively, and *G* = G*/*V*_π_ *P*_max_ is the net gain. The structure of the matrix *W* is defined by the interplay of delay time τ and the clock cycle *T* as we discussed in the section “Time Multiplexing in Delay-Based Reservoirs” above, while the matrix *W* ^*I*^ is filled randomly with uniformly distributed entries in the [−1, 1] interval.

### Delayed Feedback Tuning Using FPGA

Delayed feedback’s FPGA was operating in the finite impulse response (FIR) filter regime

11 $$x^{\prime }\left(l\right)=\overset{L-1}{\underset{i=0}{\sum }}h_{i}x\left(l-i\right)$$

where *x*(*l*) and *x* ^’^(*l*) are the input and the output delayed samples, *h*_*i*_ are the filter’s tap coefficients, *L* is the filter order. FPGA operated at either sampling rate *r* = 3.906 MHz or *r* = 976.6 kHz. The filter order *L* was varied in the range *L* ∈ [2, 232] at *r* = 3.906 MHz and in the range *L* ∈ [2,464] at *r* = 976.6 kHz. All FIR filter coefficients but the last were set to zero, while the last coefficient was set to 1, ensuring the delay time τ = *L*/*r*. By varying the FIR filter order *L* we tuned the delay time in the range [0.5, 60] μs and [2, 475] μs at the sampling rate 3.906 MHz and 976.6 kHz, respectively.

### Data Injection and Acquisition

Reservoir transient responses were digitized using the Moku:Go’s built-in datalogger operating at a sampling rate of *f* = 488.3 kHz equal to the 1/8 or 1/2 of the sampling rate of the delayed feedback. The time separation θ between the virtual neurons was set to 1/*f* so that each neuron corresponded to one sample in the data log. The readout training was performed in software using routines from the *reservoirpy* library.^39^ To synchronize the reservoir’s readout with the input signal, we employed an external function generator, producing a trigger signal that triggered bursts on the AWG and started data acquisition on the Moku:Go’s datalogger. Individual inputs were concatenated in batches to speed up the reservoir optimization, so waveforms filled all the available AWGs’ memory.

## Supplementary Material



## Data Availability

The data supporting this study’s findings are available from the corresponding author upon reasonable request.

## Notes

Cobweb diagrams and fixed-point analyses of the optoelectronic oscillator; schematic and design of the electro-optic modulator driver circuit (PDF)
