# Supplementary material for: In-Situ Optimization of an Optoelectronic Reservoir Computer with Digital Delayed Feedback
Source: ACS Photonics. 2025 Jul 11;12(9):5097–105. doi: 10.1021/acsphotonics.5c01056 (PMC12447701; doi:10.1021/acsphotonics.5c01056)
Supplement: Supplementary file 1 [file ph5c01056_si_001.pdf]

Supporting Information

In-Situ Optimization of Optoelectronic Reservoir Computer with Digital Delayed Feedback

Fyodor Morozko, Shadad Watad, Amir Naser, Antonio Calà Lesina, Andrey Novitsky, Alina Karabchevsky

Number of pages: 4
Number of figures: 2
Number of tables: 0

Supplementary Material: In-Situ Optimization of Optoelectronic Reservoir Computer with Digital Delayed Feedback

*Fyodor Morozko Shadad Watad Amir Naser Antonio Calà Lesina Andrey Novitsky Alina Karabchevsky**

F. Morozko, S. Watad, A. Naser, A. Karabchevsky
School of Electrical and Computer Engineering
Ben-Gurion University of the Negev
Beer-Sheva 8410501, Israel
Department of Physics
Lancaster University
LA1 4YB, United Kingdom
E-mail: alinak@bgu.ac.il
A. Novitsky
Belarusian State University
Minsk, 220030, Belarus
A. Calà Lesina
Hannover Centre for Optical Technologies, Institute for Transport and Automation Technology (Faculty of Mechanical Engineering), and Cluster of Excellence PhoenixD, Leibniz University Hannover, Hannover, 30167, Germany

1 Dynamics of the optoelectronic oscillator

Figure S1 presents the behavior of the optoelectronic oscillator: Figures (a-c) show cobweb diagrams in stable, periodic, and chaotic regimes, respectively, Figures d-e show simulated and experimentally obtained fixed points in the systems at different settings based on the Ikeda model as described in Refs. [3, 1, 2, 5, 4].

As discussed in [5], if the response time of the system τ is much less than the delay time τ_D $\tau \ll \tau_D$, the continuous-time dynamics of the delayed-feedback system can be efficiently modeled by the discrete-time difference equation

$$x_{n+1} = G/2\left(1 + M\sin(\pi(x_n + x_b))\right), \tag{1}$$

where $x_n = V(n\tau_D)/V_\pi$, $x_b = V_B/V_\pi$, and

$$G = P_{\text{out}}G^*/V_\pi \tag{2}$$

is the net gain of the open loop. The graphical solution of the 1 is depicted in Figure S1. The resulting path originates from x_0 in the proximity of x_1 and moves away from x_1 due to instability. However, it is eventually attracted to a stable limit determined by the number of periods. For Figure S1a, the period is 1, for Figure S1b it is 2, and for Figure S1c, the period is 3.

The simulation results of the optoelectronic oscillator are represented in subplots Figures S1a-c, which show the cobweb diagrams of the different regimes. The method involves overlapping the cobweb plot with the function $y = x$ to identify the fixed points. Figure S1a shows the dynamics of the system in the stable regime corresponding to the parameters $P_{\max} = 0.3$ mW denotes the maximum power, $V_{\text{b}} = 0$ V represents the bias voltage, $G = 0.56$ signifies the feedback gain, and $M = 0.983$ is defined as the modulation factor. So, in the intersection, we obtain one unstable fixed point. Figure S1b shows the periodic regime with $P_{\max} = 0.5$ mW, $V_{\text{b}} = 0$ V and $G = 0.93$. Here, we encounter two unstable fixed points. The chaotic regime is shown in Figure S1c with $P_{\max} = 0.9$ mW and $V_{\text{b}} = 0$ V, $G = 1.49$. By examining the three subplots, we can observe that the stability of $x_{\text{n+1}}$ vs. x_{n} is decreasing compared to the stable regime in Figure S1a.

Hopf bifurcations are shown in Figures S1d-f. Where Figure S1d demonstrates the equilibrium values of the first iteration "N=1" for the "Number of the equation" considering the bias voltage. The black curve

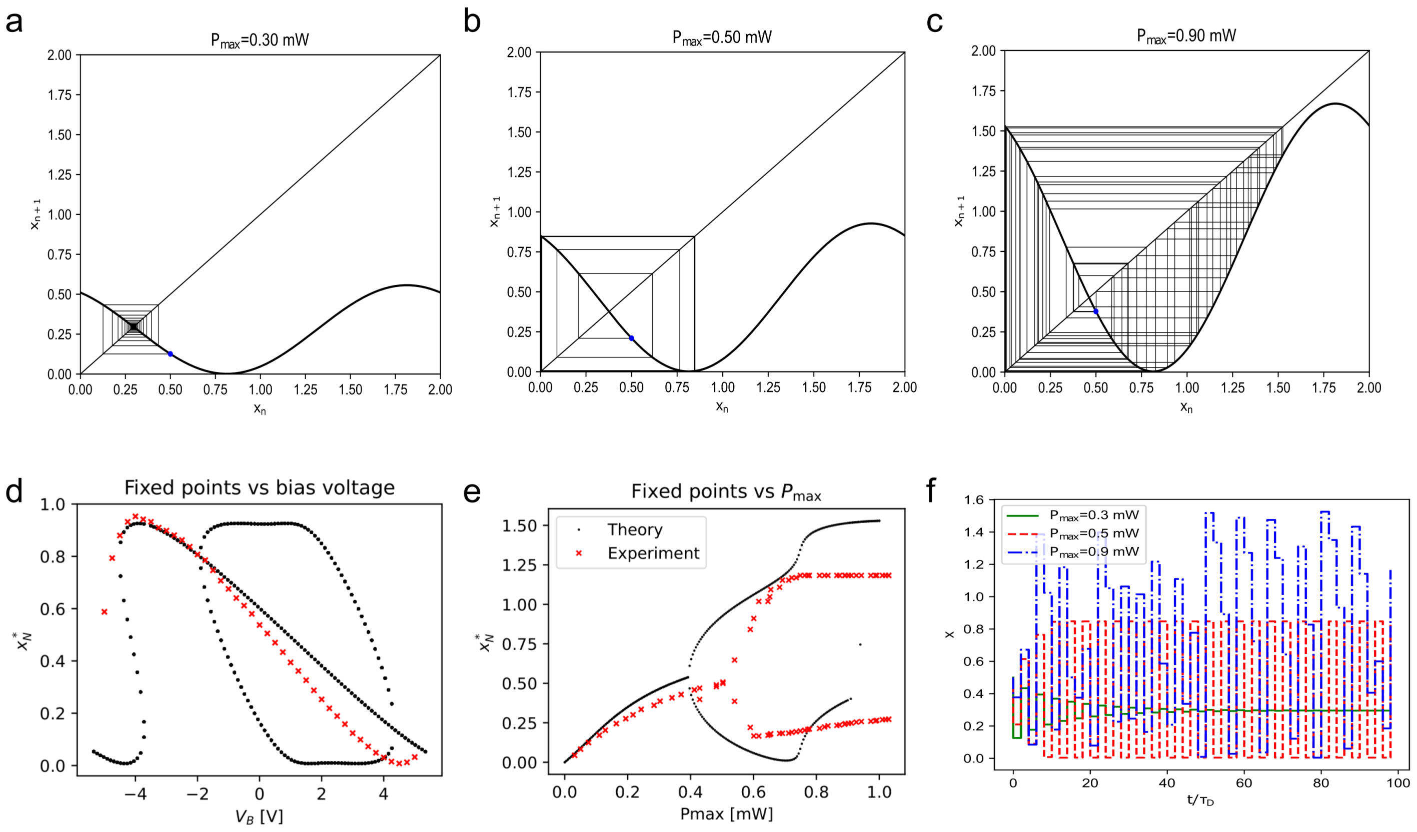

Figure S1: Simulated and experimental results from the set-up shown in Figure 2 in the main text: calculated results based on the discrete-time model: (a) stable, (b) periodic, and (c) chaotic regimes. (d-e) Fixed points of the system as a function of (d) bias voltage V_B and (e) optical power $P_{\max}$, f) Simulated dynamics using discrete-time model

represents the simulated results, while the red curve represents the experimental measurements, considering the parameters $G = 0.93$ and $M = 0.983$. The graph showcases stable regions with a single stable solution, denoting system stability. In contrast, the bistable and periodic regions exhibit three solutions: two stable and one unstable. The presence of periodic solutions arises from the equation's bifurcation of stable states. Simulated results are used to validate the theoretical predictions, ensuring the accuracy and reliability of the findings. Figure S1e, which exhibits a cascade of periodic solutions with fixed parameters $G = 0.93$ and $M = 0.983$. The graph presents a depiction of the stable and unstable fixed points as a function of the maximum power, $P_{\max}$, up to iteration $N = 8$. A significant observation is that as $P_{\max}$ increases, so does the net feedback gain, G, leading to a growing number of bifurcations. Notably, this cascade of periodic solutions appears to extend infinitely until reaching the critical value G_c=1.49. Upon reaching it, the system transforms into an aperiodic(chaotic) regime, resulting in the absence of periodic solutions. Figure S1f depicts stable, periodic, and chaotic regimes in the time domain.

2 Electro-optic modulator driver

To drive the electro-optical modulator, which has 50 Ω characteristic impedance, the low-current output signal of the Moku:Go's FIR filter needed to be amplified. For this purpose, we have built a driver circuit based on a high-speed analog operational amplifier (LM7171, Texas Instruments) as shown in Figure S2. We have incorporated a voltage divider at the input of the amplifier to adjust the relative strengths of the delayed feedback and input signal.

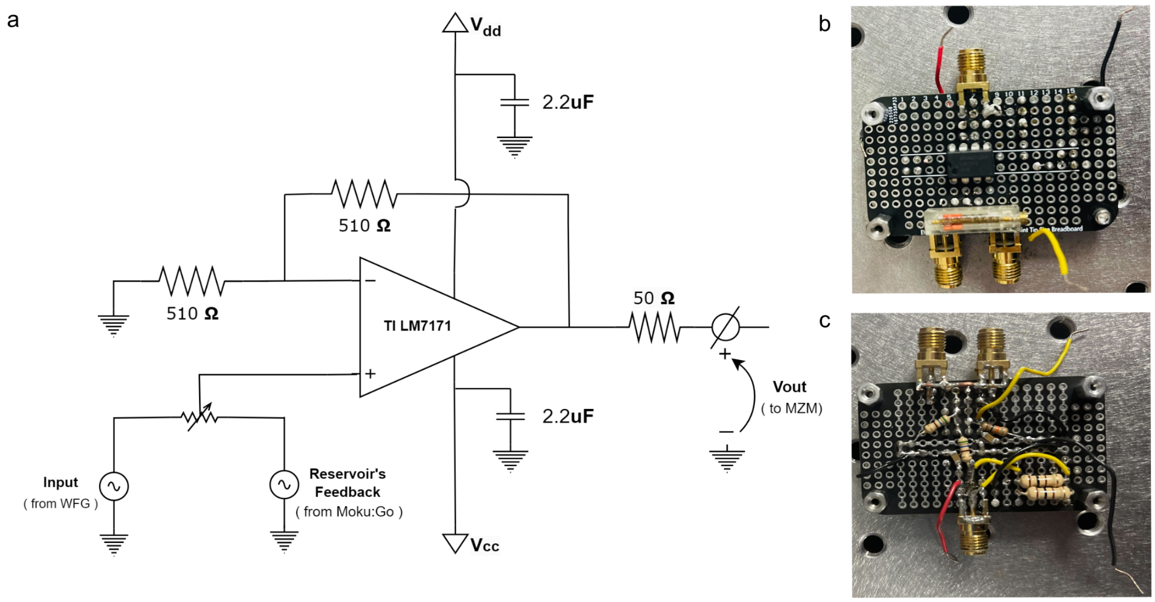

Figure S2: (a) Schematic of the electro-optic modulator driver. (b-c) design of the amplifier board.

References

[1] K. Ikeda, H. Daido, and O. Akimoto. Optical Turbulence: Chaotic Behavior of Transmitted Light from a Ring Cavity. *Physical Review Letters*, 45(9):709–712, September 1980.

[2] K. Ikeda, K. Kondo, and O. Akimoto. Successive Higher-Harmonic Bifurcations in Systems with Delayed Feedback. *Physical Review Letters*, 49(20):1467–1470, November 1982.

[3] Kensuke Ikeda. Multiple-valued stationary state and its instability of the transmitted light by a ring cavity system. *Optics Communications*, 30(2):257–261, August 1979.

[4] Laurent Larger, Jean-Pierre Goedgebuer, and Vladimir Udaltsov. Ikeda-based nonlinear delayed dynamics for application to secure optical transmission systems using chaos. *Comptes Rendus Physique*, 5(6):669–681, July 2004.

[5] A. Neyer and E. Voges. Dynamics of electrooptic bistable devices with delayed feedback. *IEEE Journal of Quantum Electronics*, 18(12):2009–2015, December 1982.
